# Cerebrovascular Resistance in Healthy Aging and Mild Cognitive Impairment

**DOI:** 10.3389/fnagi.2019.00079

**Published:** 2019-04-12

**Authors:** Larissa McKetton, Melanie Cohn, David F. Tang-Wai, Olivia Sobczyk, James Duffin, Kenneth R. Holmes, Julien Poublanc, Kevin Sam, Adrian P. Crawley, Lashmi Venkatraghavan, Joseph A. Fisher, David J. Mikulis

**Affiliations:** ^1^Joint Department of Medical Imaging, University Health Network (UHN), Toronto, ON, Canada; ^2^Krembil Brain Institute, University Health Network (UHN), Toronto, ON, Canada; ^3^Department of Psychology, University of Toronto, Toronto, ON, Canada; ^4^Department of Medicine, Division of Neurology, University of Toronto and the University Health Network Memory Clinic, Toronto, ON, Canada; ^5^Department of Physiology, University of Toronto, Toronto, ON, Canada; ^6^Institute of Medical Science, University of Toronto, Toronto, ON, Canada; ^7^Russell H. Morgan Department of Radiology and Radiological Science, The Johns Hopkins University School of Medicine, Baltimore, MD, United States; ^8^Department of Anaesthesia and Pain Management, University Health Network (UHN), Toronto, ON, Canada

**Keywords:** cerebrovascular resistance, cerebrovascular reactivity (CVR), aging, carbon dioxide, mild cognitive impairment (MCI), vascular health

## Abstract

Measures of cerebrovascular reactivity (CVR) are used to judge the health of the brain vasculature. In this study, we report the use of several different analyses of blood oxygen dependent (BOLD) fMRI responses to CO_2_ to provide a number of metrics of CVR based on the sigmoidal resistance response to CO_2_. To assess possible differences in these metrics with age, we compiled atlases reflecting voxel-wise means and standard deviations for four different age ranges and for a group of patients with mild cognitive impairment (MCI) and compared them. Sixty-seven subjects were recruited for this study and scanned at 3T field strength. Of those, 51 healthy control volunteers between the ages of 18–83 were recruited, and 16 (MCI) subjects between the ages of 61–83 were recruited. Testing was carried out using an automated computer-controlled gas blender to induce hypercapnia in a step and ramp paradigm while monitoring end-tidal partial pressures of CO_2_. Surprisingly, some resistance sigmoid parameters in the oldest control group were increased compared to the youngest control group. Resistance amplitude maps showed increases in clusters within the temporal cortex, thalamus, corpus callosum and brainstem, and resistance reserve maps showed increases in clusters within the cingulate cortex, frontal gyrus, and corpus callosum. These findings suggest that some aspects of vascular reactivity in parts of the brain are initially maintained with age but then may increase in later years. We found significant reductions in all resistance sigmoid parameters (amplitude, reserve, sensitivity, midpoint, and range) when comparing MCI patients to controls. Additionally, in controls and in MCI patients, amplitude, range, reserve, and sensitivity in white matter (WM) was significantly reduced compared to gray matter (GM). WM midpoints were significantly above those of GM. Our general conclusion is that vascular regulation in terms of cerebral blood flow (CBF) responsiveness to CO_2_ is not significantly affected by age, but is reduced in MCI. These changes in cerebrovascular regulation demonstrate the value of resistance metrics for mapping areas of dysregulated blood flow in individuals with MCI. They may also be of value in the investigation of patients with vascular risk factors at risk for developing vascular dementia.

## Introduction

The regulatory ability of the cerebral vasculature can be assessed by using a carbon dioxide (CO_2_) challenge and measuring the cerebral blood flow (CBF) response with a technique such as blood oxygen dependent (BOLD) magnetic resonance imaging (MRI). The change in BOLD response relative to the change in CO_2_ stimulus is the cerebrovascular reactivity (CVR; Mandell et al., 2008).

There are several ways of analyzing the BOLD response and presenting CVR depending on the pattern of stimulus used. A continuous regular change in stimulus from hypocapnia to hypercapnia (ramp) includes the full extent, and pattern of the flow response. A ramp stimulus also eliminates speed of response effects, because it is applied gradually enough to allow the full response to develop, even for slowly responding regions. In a step stimulus, delays in flow responses result in a decreased slope of CVR measured as a linear fit of Δ BOLD/Δ end-tidal PCO_2_ (PETCO_2_) (Poublanc et al., 2015). However, a linear fit of the BOLD response to a ramp PETCO_2_ stimulus ignores the nonlinear sigmoidal aspect of the response. In healthy subjects, a linear fit may be an acceptable CVR metric, but fitting a sigmoid to the response provides a more exact response description (Bhogal et al., 2014; Sobczyk et al., 2014). However, in patients with a compromised cerebral vasculature, BOLD responses to a PETCO_2_ ramp may take on four distinct patterns; linear increase, linear decrease (steal), U-shaped and inverted U-shaped (Fisher et al., 2017). These patterns of BOLD signal reflect the changes in flow but underlying changes in the vascular *resistance* based on simple hemodynamics (R = P/Q) cannot be assumed. In the interconnected syncytium of the cerebral vasculature, changes in perfusion pressure, and hence flow, in a voxel in response to a vasoactive stimulus is a complex function of the net changes in perfusion pressures in the surrounding voxels. With such nonlinear responses a more sophisticated method of analysis for underlying *resistance*—the fundamental parameter of the vascular physiology is needed.

**Figure 1:**
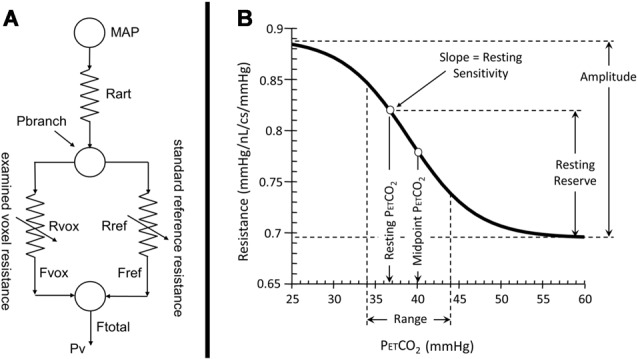
**(A)** The 2-branch vascular resistance model used to convert voxel blood oxygen dependent (BOLD) vs. PETCO_2_ to voxel resistance vs. PETCO_2_. **(B)** The sigmoidal change of vascular resistance as a function of PETCO_2_ showing the various sigmoid parameters. Abbreviations: MAP = mean arterial pressure, Rart = major cerebral arteries resistance, Pbranch = voxel perfusion pressure, Rvox = examined voxel resistance, Rref = reference voxel resistance, Fvox = examined voxel blood flow, Fref = reference voxel blood flow, Ftotal = total blood flow through both branches, Pv = venous pressure.

Consequently, a resistance model approach was developed for this purpose from one previously used to explain the cerebrovascular steal phenomenon (Figure 1A). The model consists of two vascular beds perfused from the same supply artery (Sobczyk et al., 2014). A global PETCO_2_ stimulus causes vasodilation in both branches, and consequently a drop in branch pressure because the total flow reaches the branches *via* major cerebral arteries with significantly higher resistance than those of other organs (Faraci and Heistad, 1990). If one branch cannot vasodilate, then flow falls because branch pressure falls and steal may result. This model not only explains the steal phenomenon but also shows that any mismatch of responses between the two branches will alter the distribution of flow between them.

If one branch of this model is assigned to a fixed sigmoidal resistance response to CO_2_ to act as a standard healthy tissue reference, then the resistance response to CO_2_ in the other branch can be calculated from the observed BOLD responses to CO_2_ (Duffin et al., 2017). Calculating all voxel resistance responses to CO_2_ in this way shows *they are all sigmoidal*, no matter what the pattern of the BOLD response to CO_2_ (Fisher et al., 2017). Using a standard resistance response in one branch, the calculated voxel-wise sigmoidal resistance responses to CO_2_ in the other branch are all comparable, and therefore maps of the resistance sigmoid parameters may be made. These maps provide detailed insight into the physiology and pathophysiology of vascular regulation in different brain regions not available from simply fitting the BOLD responses to CO_2_. Indeed, the resistance response sigmoids better describe the innate vascular regulatory abilities of a region, not confounded by the network flow interactions from contiguous and remote vascular beds that result during a global CO_2_ stimulus.

The parameters that describe the sigmoidal resistance responses to CO_2_ are illustrated in Figure 1B and listed as follows:

1. Amplitude, the extent of the resistance change with PETCO_2_ from maximum vasoconstriction in hypocapnia to maximum vasodilation in hypercapnia.
2. Midpoint, the PETCO_2_ of the resistance sigmoid inflexion point where the slope (sensitivity) is maximum.
3. Range, the span of PETCO_2_ stimulus over which the resistance response sigmoid is approximately linear.
4. Resting Reserve, the extent of resistance change from that at resting PETCO_2_ to maximum vasodilation.
5. Resting Sensitivity, the slope of the resistance sigmoid at resting PETCO_2_.

While maps of these resistance sigmoid parameters extend the insight into cerebrovascular regulation provided by CVR and show regions where responses may be compromised, they do not indicate how abnormal these regions may be. In order to display maps showing the extent of abnormality, a z-map approach was used (Sobczyk et al., 2015). This approach permits comparisons of resistance sigmoid parameters between different age groups and between different pathologies. For the z-map approach, an atlas of the mean and standard deviation in a voxel for each resistance metric was established from measurements in the different age groups of the control subjects, and also in the group of mild cognitive impairment (MCI) patients.

MCI in many manifests as the prodromal phase of Alzheimer’s disease (AD) which is associated with early cognitive symptoms that do not significantly affect instrumental activities of daily living, and are not severe enough to meet the criteria for dementia (Petersen, 2004). Reduced CBF in MCI patients is comparable to that in AD patients (Leijenaar et al., 2017), and is demonstrable several years prior to clinical AD diagnosis (Kelly-Hayes et al., 1995). These findings suggest that vascular dysfunction may play an important role early in the disease; a role that requires further investigation. Accordingly, this study is the first to assess cerebrovascular regulation based on a physiological explanation of the patterns of the BOLD response to a ramp PETCO_2_ stimulus in terms of the underlying changes in vascular resistance in healthy control subjects and in MCI patients. Additionally, this article presents atlases for each of the sigmoid parameters of the resistance response to CO_2_ in different healthy age groups, enabling quantitation of the degree to which MCI patients differ from controls.

## Materials and Methods

### Ethics Declaration and Subject Information

This study was approved by the Research Ethics Board of the University Health Network. Written informed consent was obtained from all subjects. We recruited 51 healthy control volunteers between the ages of 18–83 by advertisement and word of mouth. Each subject was in good health, a non-smoker, not taking medication, and denied any neurological history. PETCO_2_ data, CVR analyses including transfer function analysis (gain, phase and coherence) and the speed and magnitude of the BOLD responses to CO_2_ were previously published for these controls (McKetton et al., 2018). The MCI subjects were recruited through the University Health Network Memory Clinic at the Toronto Western Hospital. MCI patients were diagnosed by a behavioral neurologist (DT-W) following a multi-disciplinary assessment and are in accordance with the 2011 guidelines set out by the National Institute on Aging and Alzheimer’s Association consensus panels (Albert et al., 2011; McKhann et al., 2011). In addition, diagnosis of MCI was confirmed with the Clinical Dementia Rating (CDR) scale, where all MCI subjects scored 0.5 (cognitive complaint but preserved independence with instrumental activities of daily living).

The inclusion criteria for MCI recruitment included patients over the age of 50, and a diagnosis of MCI of amnestic subtype. The exclusion criteria for MCI patients included any medical contraindications to controlled hypercapnia, a diagnosis of mixed AD and vascular dementia, a history of stroke or transient ischemic attack (TIA), uncontrolled hypertension, dyslipidemia or diabetes, history of unrelated neurological disease, pulmonary disease, and any medications known to interfere with CVR measurements.

Five out of the 51 healthy control subjects and 3 out of 16 MCI subjects were excluded from the atlas creation due to image noise and unusable data; for example, an insufficient decrease of PETCO_2_ during the hypocapnic part of the ramp stimulus. One control subject was excluded due to a previous history of stroke. As such, 47 healthy control and 13 MCI subjects were included in the analyses. Four healthy control age cohorts were used for the construction of the atlas maps for comparison with one another and with the MCI group. The demographics for each subject, and Mean (SD) resting PETCO_2_ are presented in Table 1. More detailed background information in MCI and the oldest control cohort are included in the Supplementary Table S1. Performance of the MCI group and of a subset of the healthy control participants on The Toronto Cognitive Assessment (TorCA; Freedman et al., 2018), which was administered within 1 month of MRI scanning, is reported in the Supplementary Table S2.

**Table 1:** Summary of subject demographics.

Age range	*N*	Sex	Mean PETCO_2_ mmHg
18–28 (CON)	14	10 F, 4 M	39.1 (3.4)
29–38 (CON)	11	9 F, 2 M	38.6 (3.1)
39–54 (CON)	11	3 F, 8 M	38.4 (3.7)
55–83 (CON)	11	2 F, 9 M	38.1 (3.8)
61–83 (MCI)	13	8 F, 5 M	36.2 (2.8)
*Total*	60	32 F, 28 M	38.1 (1.1)

### Experimental Protocol Data Acquisition

All subjects were scanned on a 3-Tesla GE system MRI scanner (Signa HDx platform, GE Healthcare, Milwaukee, WI, USA), using an eight-channel phased array head coil at Toronto Western Hospital.

Each subject had a whole-brain high-resolutionT1-weighted 3D spoiled gradient echo (FAST-SPGR) sequence acquired first with the following parameters: TI = 450 ms, TR = 7.88 ms, TE = 3 ms, flip angle = 12°, voxel size = 0.859 × 0.859 × 1 mm, matrix size = 256 × 256, 146 slices, field of view = 24 × 24 cm, no interslice gap.

After the anatomical scan, a BOLD fMRI T2*-weighted echoplanar imaging gradient echo (EPI-GRE) sequence was acquired with the following parameters: TR = 2400 ms, TE = 30 ms, flip angle = 70°, 41 slices, voxel size = 3.5 mm^3^, matrix size = 64 × 64, number of frames = 335, field of view = 24 × 24 cm.

### Test Protocol

**Figure 2:**
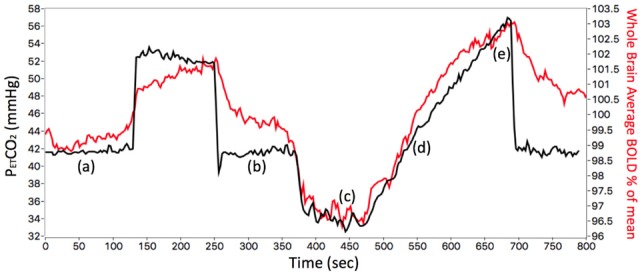
The stimulus sequence for a representative control subject (ID 26, F, 38). The PETCO_2_ stimulus (black line) and whole brain average BOLD voxel response (red line) are shown. The first part of the stimulus, between points (a) and (b), consisted of a step change used to measure time delay reported previously (12). The subject’s resting PETCO_2_ of 41 mmHg (a) was increased by 10 mmHg for 2 min then returned to resting PETCO_2_ (b). The ramp portion shown between points (c) and (e) was used for assessing the resistance response. The subject’s resting PETCO_2_ was decreased by 10 mmHg (c) and then slowly increased to resting PETCO_2_ (d), further increased to ~15 mmHg above resting (e), and returned to resting PETCO_2_.

PETCO_2_ and end-tidal PO_2_ (PETO_2_) were controlled using an automated computer-controlled gas blender that regulated gas composition and flow to a sequential gas delivery breathing circuit (RespirAct^TM^, Thornhill Research Inc., Toronto, ON, Canada; Slessarev et al., 2007; Fierstra et al., 2013; Fisher, 2016). The RespirAct^TM^ was connected to a soft plastic mask that subjects breathed through in the scanner and was sealed to their face using transparent dressing film (Tegaderm Film, 1626W, #M Health Care, St. Paul, MN, USA). PETCO_2_ was tightly controlled during rest by targeting each subject’s resting PETCO_2_ [mean (SD) 38.8 (3.4); range 30–45 mmHg], and PETO_2_ was targeted at 100 mmHg throughout. The PETCO_2_ stimulus design was programmed into the RespirAct^TM^ that ran the prospective gas-targeting algorithm (Slessarev et al., 2007), that allowed the CO_2_ stimulus to be controlled such that PETCO_2_ was equivalent to PaCO_2_ (Ito et al., 2008; Willie et al., 2012). The stimulus consisted of a 13.4-min sequence that started with the clamping of each subject’s baseline PETCO_2_ for ~2 min, followed by a 2-min step increase by 10 mmHg that returned back to baseline for 2 min. This was followed by a slow decrease in PETCO_2_ by 10 mmHg over 30 s that was followed by a steady increase in PETCO_2_ to ~15 mmHg above baseline ~5 min, and a return to baseline for ~2 min (Figure 2). The blood flow response to CO_2_ was measured using BOLD as this is a reasonable surrogate of CBF within the range of blood flow changes induced by the applied CO_2_ stimuli (Hoge et al., 1999).

### Data Analyses

MRI and PETCO_2_ data were transferred to an independent workstation and preprocessed using AFNI software (National Institutes of Health; Cox, 1996), MATLAB R2015a^1^ and SPM8; Wellcome Department of Imaging Neuroscience, University College, London, UK^2^). Functional BOLD images were volume registered, slice-time corrected, and co-registered to the anatomical T1-weighted scan. PETCO_2_ data were re-sampled and time-shifted to the point of coincidence between the rapid changes in PETCO_2_ and BOLD signal. An experienced neuroradiologist (DM) screened for white matter (WM) hypointensities on T1-weighted images in the older cohort to ensure that any group differences in resistance metrics were not due to results of group differences in ischemic small vessel disease. The T1 weighted images were segmented into CSF, gray matter (GM) and WM using SPM8 (Wellcome Department of Imaging Neuroscience, Institute of Neurology, University College, London, UK). These generated probability maps of GM and WM that were thresholded at 70% probability and then transformed into Montreal Neurological Institute (MNI) space. GM and WM masks were combined and applied for threshold-free cluster enhancement (TFCE) and assessment of mean and ramp CVR for group comparison.

The resistance model analysis calculations were performed using a custom program LabVIEW (National Instruments, Texas). The model calculations producing the resistance maps make four major assumptions. (1) brain metabolism remains constant and neural activation is unchanged during the ramp PETCO_2_ stimulus from hypo- to hypercapnia under resting conditions; (2) BOLD signals represent the *pattern* of the flow response to CO_2_; (3) both the resistance of the supply artery (Rart) and mean arterial pressure (MAP) in the model are fixed and unchanged with CO_2_; and (4) resistance response patterns to the ramp PETCO_2_ are sigmoidal in that they are at their maximum during hypocapnia and are at their minimum during hypercapnia (Duffin et al., 2017). Resistance units in the model are relative only and were arbitrarily set at (pressure/flow, mmHg/nL/s); (5) The reference branch resistance variation with PETCO_2_ was fixed as:

(1) $$\text{Reference Standard Resistance = 0.75 }-\text{0.12/}\left(1+{\text{e}}^{-\left({\text{(PETCO}}_{2}-40)/4.5\right)}\right)$$

Resistance maps were created from each voxel’s BOLD response to the PETCO_2_ ramp stimulus. The BOLD response for every voxel became the examined branch flow in the model, and its resistance sigmoid parameters were calculated (Figure 1) using a constrained Levenberg-Marquardt fitting algorithm. The fitting constraints were 0.6–1 mmHg/nL/s for the minimum resistance in hypocapnia, −0.3 to −0.001 mmHg/nL/s for amplitude, 20–60 mmHg PETCO_2_ for midpoint, and 0.1–10 mmHg PETCO_2_ for range. If any voxel parameter reached one of these fitting constraints, that voxel was set to an average of the parameters in the surrounding voxels that met the constraints. In this way, a number of resistance maps were generated for each subject to generate atlas maps for every age cohort.

In order to calculate resting reserve and sensitivity (Figure 1B), each subject’s resting PETCO_2_ was determined as follows. First, it was assumed that the midpoint of the resistance sigmoid calculated for healthy voxels was that subject’s resting PETCO_2_. Voxels considered to be of healthy tissue were those with CVR values within the range from 0.25 to 0.35% BOLD/mmHg, and with an *r*^2^ fit > 0.7. Then a mean resistance sigmoidal response to CO_2_ for these healthy voxels was calculated and its midpoint found and used as the subject’s healthy resting PETCO_2_.

Voxel resistance sigmoid amplitude maps show the voxel resistance sigmoid amplitude, while the midpoint resistance maps show the PETCO_2_ (mmHg) of the resistance sigmoid midpoint. The resistance range maps show the range of PETCO_2_ (mmHg) over which the resistance response sigmoid can be considered linear. Resting reserve maps display the vasodilatory change in resistance from that at the healthy resting PETCO_2_ to that in hypercapnia, and resting sensitivity maps show the slope of the resistance sigmoidal response to CO_2_ at the healthy resting PETCO_2_.

### Atlas Map Generation

Each subject’s anatomical T1-weighted IR-FSPGR brain volume was co-registered into MNI (Montreal Neurological Institute, Montréal, QC, Canada) standard space using the T1-weighted MNI152 standard template (Ashburner and Friston, 1999) with a 12-parameter affine transformation (Ashburner and Friston, 1997) and nonlinear deformations. The resulting transformation was applied to the analyzed BOLD data for each subject using a 5-mm full-width half-maximum (FWHM) spatial smoothing kernel at each voxel. Both the mean (μ) and standard deviation (σ) for the CVR in each voxel was calculated in AFNI. Atlas maps, whose creation has been further detailed elsewhere (Sobczyk et al., 2015), were then generated for μ and the coefficient of variation (σ/μ). Resistance atlas maps were created and compared between each age cohort, and the MCI group.

TFCE analysis was performed on the data. This approach was used in order to enhance cluster-like structures and has been shown to provide better sensitivity than other methods as previously described (Smith and Nichols, 2009). The ROI mask used for TFCE analyses consisted of the combined GM and WM with the CSF removed. Permutation testing was then applied to the height of the maxima of the resulting statistic image, using the “randomize” permutation-based inference tool (Winkler et al., 2014) in FSL v.5.0.9 (FMRIB Library^3^) that allowed for the maintenance of strong control over family-wise error.

Additional analyses were conducted on the mean GM and WM resistance metrics for amplitude, midpoint, range, reserve, and sensitivity. Statistical analyses were run using IBM SPSS Statistics (IBM SPSS Statistics for Macintosh, Version 21.0, Armonk, NY, USA). For each resistance metric, a mixed model analysis of covariance (ANCOVA), with MCI (61–83) and control cohort age group (18–28, 29–38, 39–54, and 55–83) as the between-groups variable and GM and WM for each resistance metric as the within-group variable, using sex as a covariate was run. Results were considered significant and accounted for multiple comparisons by Bonferroni correction if the per-comparison *p*-value was less than 0.05/(2 comparisons) = 0.025. Data for all tests were normally distributed as confirmed by the Shapiro-Wilk test (*p* > 0.05).

Further analyses were done to see how resistance sigmoids differ between vascular territories in healthy controls. Manually traced regions of interest (ROIs) identified by two neuroradiologists (DJM and DMM) were used for each major arterial vascular territory; the anterior cerebral artery (ACA), middle cerebral artery (MCA), and posterior cerebral artery (PCA). For each resistance metric, a mixed model ANCOVA was run using the entire age cohort (18–83) as the dependent variable, using each vascular territory (ACA, MCA, and PCA) and matter type (GM, WM) as the independent variables, using sex as a covariate. Results were considered significant and accounted for multiple comparisons by Bonferroni correction if the per-comparison *p*-value was less than 0.05/(6 comparisons) = 0.0083.

## Results

### PETCO_2_ Results

Resting PETCO_2_ values were previously reported in 51 controls with the mean (SD) of 38.8 (3.4) mmHg (McKetton et al., 2018). A one-way analysis of variance (ANOVA) revealed no significant differences between resting PETCO_2_ values between the MCI group and each healthy control age cohort *F*_(4,59)_ = 1.57, *p* = 0.19.

### Atlas Results

**Figure 3:**
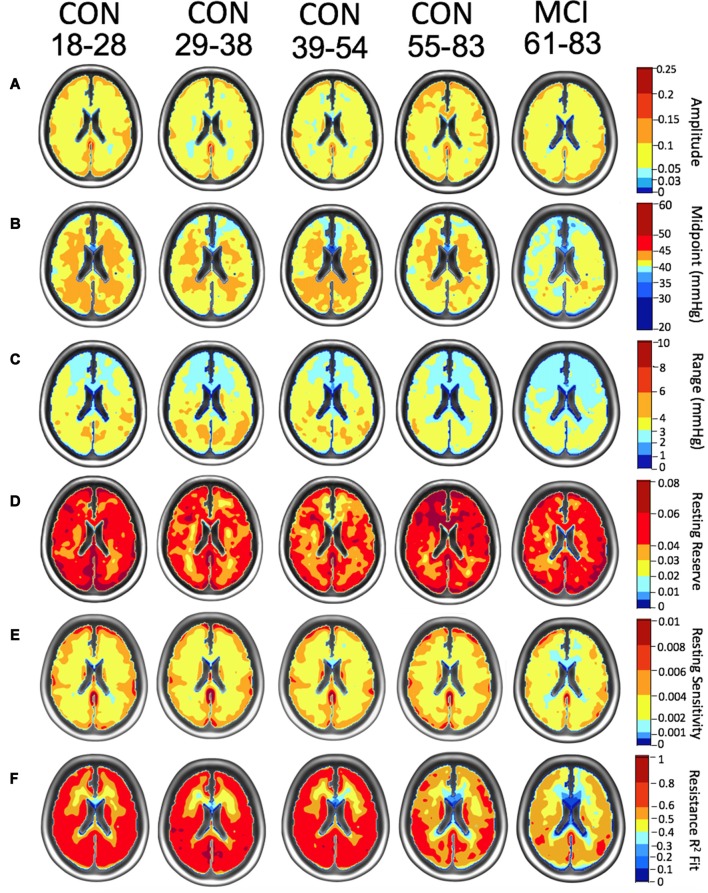
Axial atlas images of resistance parameters for each age group of the control subjects (CON) and the mild cognitive impairment (MCI) patient group are depicted using nonlinear color scales. **(A)** Resistance sigmoid amplitude maps show increasing amplitudes from dark blue to light blue then yellow to dark red. **(B)** Resistance sigmoid midpoint maps (mmHg) show midpoints above (yellow to dark red) and below (light to dark blue) the reference resistance sigmoid midpoint. **(C)** Resistance sigmoid range maps show increased (yellow to dark red) and decreased (light to dark blue) linear ranges in mmHg. **(D)** Resistance resting reserve, the vasodilatory ability from healthy resting PETCO_2_, is shown with the amplitude color scale. **(E)** Resistance resting sensitivity, the sigmoid slope at healthy resting PETCO_2_, is also shown with the amplitude color scale. **(F)** Resistance *r*^2^ fit also uses a similar color scale.

Atlas maps of the resistance sigmoid parameters were created for the control subjects in each age group and compared between one another to discern age-related differences. In addition, comparisons were made between the healthy control groups and the MCI patient group. Figure 3 shows these atlas maps. The amplitude maps show the difference between the maximum and minimum resistance values, reflecting the resistance range between the vasoconstrictive and vasodilatory bounds. The midpoint maps show the PETCO_2_, at which resistance response sensitivity is a maximum. The range maps show the PETCO_2_ range over which the resistance change with PETCO_2_ may be considered linear, the resting reserve and sensitivity maps show the ability of areas to vasodilate from healthy resting PETCO_2_, and the *r*^2^ maps show the goodness of fit of the resistance sigmoids.

TFCE analysis found a significant difference in amplitude for a 1,944 mm^3^ cluster comprising the bilateral thalamus and posterior corpus callosum between groups CON (18–83) and MCI (63–83), and in a 4,448 mm^3^ cluster comprising the bilateral temporal cortex, thalamus, anterior corpus callosum, and brain stem between groups CON (55–83) and CON (18–54), *p* < 0.05 (Figure 4.1). There was also a significant reduction in reserve in a 200 mm^3^ cluster comprising the thalamus and posterior corpus callosum in the MCI group compared to the youngest control group, and in a 2,352 mm^3^ cluster comprising the right anterior cingulate cortex, right superior frontal gyrus, and anterior and mid corpus callosum in the oldest control group CON (55–83) and the youngest control group CON (18–54), *p* < 0.05 (Figure 4.2). There was a significant reduction in range in a 180,552 mm^3^ cluster globally in the MCI group compared to all control groups, *p* < 0.05 (Figure 4.3).

**Figure 4:**
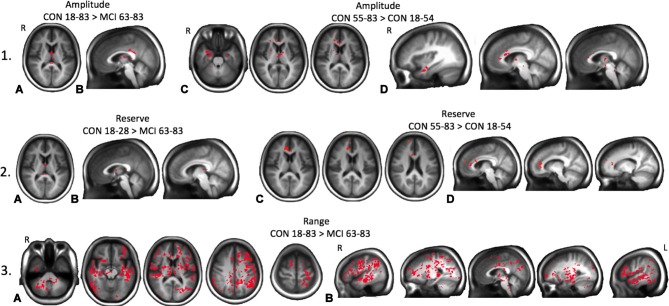
Maps of significant differences between groups for resistance amplitude, reserve, and range determined from threshold-free cluster enhancement (TFCE) analysis are shown as red clusters. **(1A)** Axial and **(1B)** sagittal views showing significantly reduced amplitudes in the MCI patient group compared to all control groups combined. **(1C)** Axial and **(1D)** sagittal views of significantly reduced amplitudes in the youngest control group compared with the oldest control group. **(2A)** Axial and **(2B)** sagittal views showing significantly reduced resting reserve in the MCI patient group compared with the youngest control group. **(2C)** Axial and **(2D)** sagittal views showing significantly reduced resting reserve in the combined younger control groups compared with the oldest control group. **(3A)** Axial and **(3B)** sagittal views showing significantly reduced resistance sigmoid range in the MCI patient group compared with all healthy control groups combined, *p*s < 0.05.

There was a significant reduction in resistance midpoints in a 14,216 mm^3^ cluster comprising GM and WM frontal regions, superior parietal cortex, posterior central cortex, right caudate and putamen in the MCI patient group compared to the oldest age-matched control group. This result was exaggerated when the MCI patient group was compared to all the control groups combined, predominantly in the right hemisphere globally, *p* < 0.05 (Figure 5.1). There was a significant reduction in resistance resting sensitivity in a 113,976 mm^3^ cluster predominantly in the right hemisphere in the MCI patient group compared with the oldest age-matched control group. This result was also exaggerated globally when comparing the MCI patient group with all the control groups combined, *p* < 0.05 (Figure 5.2).

**Figure 5:**
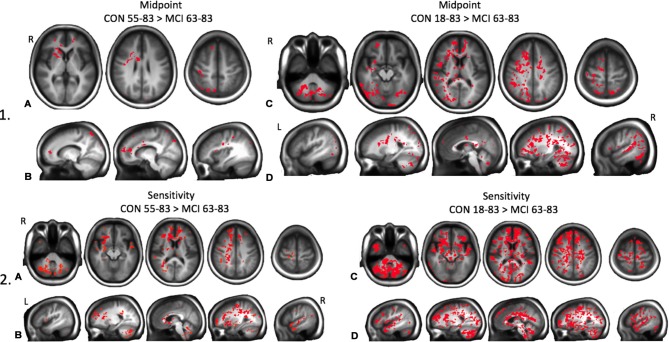
Maps of significant differences between groups for resistance sigmoid midpoints and sensitivities determined from TFCE analysis are shown as red clusters. **(1A)** Axial and **(1B)** sagittal views of significantly decreased midpoints for the MCI patient group compared to the oldest healthy control group. **(1C)** Axial and **(1D)** sagittal views of significantly decreased midpoints in the MCI patient group compared to all healthy control groups combined. **(2A)** Axial and **(2B)** sagittal views of significantly decreased sensitivity in the MCI patient group compared to the oldest healthy control group. **(2C)** Axial and **(2D)** sagittal views of significantly decreased sensitivity in the MCI patient group compared to all healthy control groups combined, *p*s < 0.05.

TFCE analysis was also applied to the resistance sigmoid *r*^2^ fit maps to detect any correlation between fit quality and significant difference in parameters. While some overlap was found in most regions of resistance parameter, TFCE analysis differences did not overlap with those of the *r*^2^. We also noted the percentage of voxels that fell within the resistance sigmoid fit constraints and found no significant differences between age groups, but a decrease in the MCI group compared to the controls.

Additional analyses were done on the mean GM and WM resistance metrics for amplitude, midpoint, range, reserve, and sensitivity in controls and MCI patients (Table 2). Statistics on these metrics are reported in (Table 3).

**Table 2:** Resistance metrics for gray matter (GM) and white matter (WM) in mild cognitive impairment (MCI) and healthy control groups.

	Amplitude (mmHg/nL/s)		Midpoint (mmHg)		Range (mmHg)		Reserve (mmHg/nL/s)		Sensitivity (mmHg/nL/s)	
Group	GM	WM	GM	WM	GM	WM	GM	WM	GM	WM
CON 18–28	0.107 (0.012)	0.077 (0.012)	40.61 (1.75)	41.69 (1.47)	3.49 (0.58)	3.26 (0.58)	0.059 (0.014)	0.046 (0.015)	0.0053 (0.0008)	0.0035 (0.0006)
CON 29–38	0.102 (0.012)	0.072 (0.010)	40.05 (1.44)	41.28 (1.37)	3.55 (0.61)	3.29 (0.50)	0.053 (0.008)	0.042 (0.009)	0.0054 (0.0008)	0.0034 (0.0006)
CON 39–54	0.100 (0.019)	0.069 (0.013)	40.87 (1.92)	41.77 (1.45)	3.42 (0.64)	3.24 (0.64)	0.052 (0.013)	0.040 (0.011)	0.0053 (0.0007)	0.0034 (0.0003)
CON 55–83	0.112 (0.019)	0.084 (0.022)	40.38 (1.25)	41.36 (1.44)	3.39 (0.42)	3.12 (0.38)	0.060 (0.016)	0.051 (0.002)	0.0054 (0.0006)	0.0036 (0.0005)
MCI 61–83	0.099 (0.022)	0.076 (0.010)	38.02 (1.80)	39.70 (1.30)	2.98 (0.66)	2.83 (0.63)	0.055 (0.011)	0.046 (0.012)	0.0044 (0.0009)	0.0029 (0.0008)

*Each resistance metric shows the mean (SD); mmHg/nL/s*.

**Table 3:** Statistics on resistance metrics for GM and WM in MCI and healthy control groups.

	Main effect of group	Group by matter type interaction	Effect of matter type (GM vs. WM)
Amplitude	*F*_(4,54)_ = 1.74, *p* = 0.15	*F*_(4,54)_ = 2.65, *p* = 0.043	*F*_(1,54)_ = 517.21 ***p < 0.0001
Midpoint	*F*_(4,54)_ = 5.18, *p* = **0.001	*F*_(4,54)_ = 3.12, *p = 0.02	*F*_(1,54)_ = 99.22, ***p < 0.0001
Range	*F*_(4,54)_ = 2.01, *p* = 0.11	*F*_(4,54)_ = 1.79, *p* = 0.15	*F*_(1,54)_ = 85.05, ***p < 0.0001
Reserve	*F*_(4,54)_ = 1.3, *p* = 0.28	*F*_(4,54)_ = 1.94, *p* = 0.12	*F*_(1,54)_ = 157.14, ***p < 0.0001
Sensitivity	*F*_(4,54)_ = 3.19, *p* = *0.02	*F*_(4,54)_ = 2.83, *p* = 0.34	*F*_(1,54)_ = 510.83, ***p < 0.0001

*Post hoc pairwise comparisons revealed significantly decreased WM amplitudes, ranges, reserves, and sensitivities, compared to GM and decreased GM midpoints compared to WM for each group *p*s < 0.0001. Post hoc pairwise comparisons revealed significantly decreased GM midpoints and resting sensitivities (*p*s < 0.01) and WM midpoints and resting sensitivities *(p*s < 0.05) in MCI compared to each control group*, where ***denotes a *p* < 0.0001.

**Figure 6:**
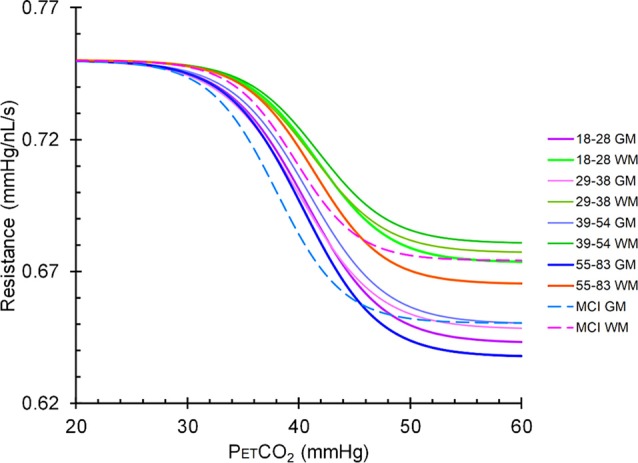
Mean resistance sigmoids for gray matter (GM) and white matter (WM) in the MCI patient group and the healthy control groups. The upper group of sigmoidal curves is in WM and the lower set are in GM. Solid lines represent the sigmoidal curves in healthy controls broken down by age cohort, and dashed lines represent the sigmoidal curves in the MCI patient group.

**Figure 7:**
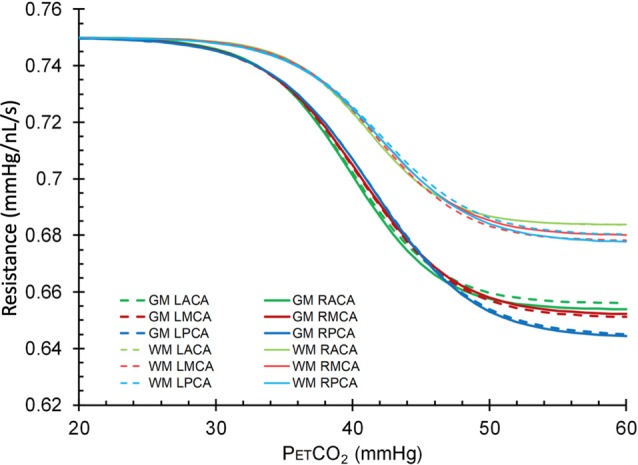
Mean resistance sigmoids for GM and WM in each vascular territory for the healthy control group. L, left; R, right, ACA, anterior cerebral artery; MCA, middle cerebral artery; PCA, posterior cerebral artery.

The resistance sigmoid parameters, amplitude, midpoint and range for each group were used to create sigmoids for both GM and WM (Figure 6). Mean resistance sigmoids for GM and WM in each vascular territory for the healthy control group were also calculated (Figure 7), and ANOVA showed that these resistance sigmoids did not differ between territories. Because BOLD is a relative measure, the calculated resistance changes with CO_2_ are also relative and resistance sigmoids cannot be compared in terms of their resting resistance. Consequently, the resistance in hypocapnia was normalized to 0.75 mmHg/nL/s for the sigmoid comparisons, and resistance was calculated as:

(2) $$\text{Resistance sigmoid value = 0.75}-\text{amplitude/}\left(1+{\text{e}}^{-\left(({\text{PETCO}}_{2}-\text{midpoint)/range}\right)}\right)$$

## Discussion

This study is the first application of a novel measure of cerebral hemodynamics based on sigmoidal vascular resistance profiles. Vascular resistance responses to CO_2_ measure the fundamental aspects of cerebrovascular regulatory physiology. The advantage of this assessment is that it is not influenced by the non-linear responses resulting from the redistribution of blood flow during a global CO_2_ stimulus, which may take on four different patterns, linear increase, linear decrease (steal), U-shaped and inverted U-shaped responses (Fisher et al., 2017).

We found significant differences in all resistance sigmoid parameters when comparing the MCI patient group to the control groups, with the greatest difference detected by both TFCE and ANCOVA analyses of decreased resistance sigmoid midpoint and sensitivity in the MCI group compared with each healthy control group. The latter finding supports the concept that MCI patients have altered CBF responses to vasodilatory demand. In contrast, with respect to aging in the healthy control subjects, we found that the resistance sigmoid parameters were not significantly changed with age until the oldest group, findings similar to those we reported previously for other CVR metrics (McKetton et al., 2018). In the oldest compared to the youngest, some resistance sigmoid parameters indicated an increased ability to regulate CBF with age, an unexpected finding.

### Resistance and Aging

CBF is regulated over a range of perfusion pressures by adjusting cerebrovascular resistance with the constriction and dilation of arterioles. The aging brain has been shown to have a decreased global CBF (Marchal et al., 1992; Catafau et al., 1996; Krausz et al., 1998; Krejza et al., 1999; Nobler et al., 1999; Schultz et al., 1999; Chen et al., 2011) at the same perfusion pressure, reduced dynamic BOLD-CBF coupling during resting state (Chiacchiaretta et al., 2018), and a gradual increase in the index of cerebral vascular resistance measured with color-coded Doppler sonography (Krejza et al., 1999). However, the increased resistance response metrics may simply reflect the increase in vascular tone in response to age-related increase in blood pressure. With regard to CVR, an age-related decrease in complexity of the neuropil or neuronal drop-out would result in an increase in BOLD signal for a given flow resulting from a reduced oxygen extraction. This is consistent with cerebral atrophy in both GM and WM volume loss, with increased volume (Resnick et al., 2003) and cortical thickness atrophy rates (Thambisetty et al., 2010) primarily in frontal and parietal regions in aging humans.

These changes are associated with reduced brain perfusion with age but CVR is required to assess the residual vasoactive regulatory ability of the cerebral vasculature.

CVR changes with age have been described previously, with many studies, using a variety of testing methods, reporting a decline with age (Schieve and Wilson, 1953; Yamaguchi et al., 1979; Yamamoto et al., 1980; Reich and Rusinek, 1989; Tsuda and Hartmann, 1989; Kastrup et al., 1998; Lu et al., 2011; Gauthier et al., 2013; Flück et al., 2014; De Vis et al., 2015; Coverdale et al., 2017; Peng et al., 2018). Other studies found no significant differences in CVR with age (Davis et al., 1983; Ito et al., 2002; Schwertfeger et al., 2006; Murrell et al., 2013; Oudegeest-Sander et al., 2014). The variety of testing methods and a lack of use of a standard stimulus for CVR testing may account for inconsistencies in these findings and led us to re-examine CVR in aging using our standard stimulus methodology. In that study (McKetton et al., 2018) we found that CVR did not decline significantly until the oldest group.

Here we report that resistance sigmoid parameters were also not significantly changed with age until the oldest group, findings similar to our previous CVR metrics (McKetton et al., 2018). However, it was therefore surprising that the oldest control group were found to have increased resistance amplitude in clusters within the temporal cortex, thalamus, corpus callosum and brainstem, and increased resting resistance reserve in clusters within the cingulate cortex, frontal gyrus, and corpus callosum compared to the younger control cohort (Figure 4). These findings suggest that vascular reactivity is initially maintained with aging and then surprisingly *increases*. We do not have a good explanation for this finding and offer the following speculative possibilities. Explanations may include: (1) the resistance response improves due to the increase in basal vascular tone responding to the increase in blood pressure with age; and (2) age-related declines in cognitive functioning somehow result in activation of compensatory neural networks located in these areas. The intensity of this compensatory activation may require increasing degrees of local blood flow support achieved through a greater degree of amplitude and reserve response to a stimulus. We considered the possibility that the resistance sigmoid fitting quality was poorest in the eldest group but could find no evidence of overlap between poor *r*^2^ fits and these areas. Furthermore, the number of voxels fitted with sigmoids within the goodness of fit constraints did not differ with age and was >80% for all age groups. This means that the fidelity of the response to the stimulus is unchanged. We are therefore confident that these findings are correct and can only suggest that they may indicate an overcompensation in these regions.

Linked to these findings are the resistance sigmoid differences between GM and WM (Table 3 and Figure 7), where for all age groups the highest amplitudes were found in GM and the lowest amplitudes in WM. We suggest that this latter difference may be due to the lower metabolic requirement and blood flow per volume of tissue in WM compared to GM (Ransom et al., 2011).

The resistance sigmoid midpoint maps indicate whether the resistance response is shifted to the left or right (to higher or lower PETCO_2_) of the reference resistance sigmoid midpoint. Blood flow regulation is most effective, with maximum sensitivity, at the midpoint, and therefore the midpoint should coincide with the resting PETCO_2_. The GM and WM midpoints were close to the healthy resting PETCO_2_, with the WM slightly shifted to the right (higher PETCO_2_).

An explanation for this may be that GM responds faster than WM (Poublanc et al., 2015), so that GM flow increases at the expense of WM flow initially, but WM then increases leading to a U-shaped response see (Fisher et al., 2017). Alternatively, the increased WM midpoint, therefore, suggests a mild adaptation in WM blood flow regulation to obtain the highest sensitivity at an elevated PCO_2_ such as could result from the lower resting blood flow in WM compared to GM. Indeed, that was found for the midpoint atlas maps in all age groups (PETCO_2_ ~40 mm Hg).

### MCI Patient Findings

As Figure 6 illustrates, the resistance sigmoid midpoints for both GM and WM were both shifted to the left in MCI compared to controls. This finding suggests that the resistance at resting PETCO_2_ is lower for MCI than controls, and although we did not measure the CBF, it has been shown to be higher at rest in MCI (Dai et al., 2009). Eventually, both the compensatory neuronal activity and associated blood flow compensation decline as neurodegeneration continues; resulting in further decreased cognition, decreased blood flow, and decreased GM volumes leading to dementia. This supposition remains debatable since regional CBF maps showing both ASL have shown both hypoperfusion and hyperperfusion patterns in MCI and AD patients compared with controls. These variations in results are difficult to interpret due to the large variations in methodology, and heterogeneity in patient demographics, dementia sub-types, and in the neuropsychological assessments used (see review, Sierra-Marcos, 2017).

In terms of the ability to regulate CBF, we note that the resistance amplitude maps provide indications of the vasodilatory and vasoconstrictive reserves relative to the resting PETCO_2_. Of particular interest, is the map of the vasodilatory reserve at the subject’s healthy resting PETCO_2_, that we termed the resting reserve map. This map provides a picture of the brain’s ability to vasodilate from rest without being confounded by complex flow redistribution between vascular territories resulting from a global CO_2_ stimulus. Resting reserve may, therefore, be one of the best identifiers of pathophysiology. It solves the dilemma of choosing the best stimulus to apply as long as the stimulus chosen includes the entire range of vasoactive responses, as we have achieved through the application of the ramp stimulus. Our TFCE results showed both decreased amplitude and reserve in the corpus callosum and thalamus in the MCI patient group compared with the control groups; a difference most pronounced when compared with the youngest control group.

Our findings suggest that early cognitive symptoms associated with MCI are linked to the brain’s reduced ability to vasodilate from rest particularly in subcortical structures such as the corpus callosum. Previous studies have shown various results. For example, patients with amnestic MCI were found to have atrophy in posterior sub-regions of the corpus callosum (Wang et al., 2006), reduced WM density in the anterior corpus callosum sub-region (Di Paola et al., 2010), and atrophy in the anterior corpus callosum in amnesic and multi-domain amnesic patients compared to healthy controls (Thomann et al., 2006). However, other studies found no callosal differences between amnestic MCI (Wang and Su, 2006) and multi-domain MCI (Hallam et al., 2008) compared to healthy controls. Additionally, the thalamus has been implicated in amnestic MCI. Compared to healthy controls, amnestic MCI patients were found to have decreased functional connectivity between the thalamus and other structures using resting-state fMRI (Wang et al., 2012; Zhou et al., 2013; Cai et al., 2015), structural abnormalities in the thalamo-cortical WM fiber pathways (Alderson et al., 2017), significant neuroanatomical thalamic shape differences (Leh et al., 2016; Hahn et al., 2016), and smaller left thalamic volumes (Hahn et al., 2016).

### Limitations

The present study had several limitations. First, a number of subjects had to be removed from resistance sigmoid analyses due to low SNR, motion artifacts, and the inability to hyperventilate enough during the hypocapnic portion of the ramp stimulus, which was needed to accurately determine the sigmoidal resistance parameters. Additionally, this study had a cross-sectional design that was limited to greater inter-subject variability. Ideally, a longitudinal assessment would be more appropriate although it would be limited due to a long acquisition time frame. We used SPM that employs a demon algorithm -DARTEL for image registration (Ashburner, 2007), although we acknowledge other methods such as the symmetric image normalization method may perform better in at-risk elderly individuals such as those with frontotemporal dementia and AD (Avants et al., 2008). While the use of BOLD as a surrogate measure of CBF is common, a number of assumptions are made in equating CBF and BOLD changes, and these have been discussed in detail in previous articles (Sobczyk et al., 2014; Duffin et al., 2015; Poublanc et al., 2015). BOLD correlates well with more direct methods of measurement such as arterial spin labeling when used to measure CVR (Mandell et al., 2008), and is a reasonable surrogate of blood flow for CVR measurement of hemodynamic impairment in patients with carotid artery stenosis or occlusion (Donahue et al., 2014). Moreover, BOLD has recently been validated against PET in these patients (Fierstra et al., 2018). Consequently, we suggest that the use of BOLD as a surrogate measure of CBF in this study is appropriate, but add the caveat that the effect of advanced aging is unknown.

## Conclusion

Our study assessed and compared resistance sigmoid parameter maps between different healthy age groups and with an MCI patient group. In general, the vascular regulation of CBF in response to CO_2_ was not significantly affected by age, although resting reserve and amplitude may have improved in sub-cortical structures in the eldest group. All sigmoidal resistance parameters were reduced in the MCI patient group compared with the control groups, with the greatest global differences occurring as a leftward shift in resistance midpoint (decreased PETCO_2_), and decreases in range and sensitivity. These findings provide new insights into the changes in vascular regulation that occur during the pathogenesis of MCI and AD. This information is important since AD is now thought to have a significant vascular component. CVR methodology may, therefore, be used in the diagnosis and management in this condition.

## Ethics Statement

This study conformed to the standards set by the latest revision of the Declaration of Helsinki and was approved by the Research Ethics Board of the University Health Network. Written informed consent was obtained for each subject in this study.

## Author Contributions

LM, JD, and DM designed the study. KS, KH, OS, and LM collected the data. LM analyzed the data and wrote the manuscript. DT-W referred and confirmed the diagnosis of MCI. MC performed the cognitive assessments. LM, DT-W, MC, OS, JD, JP, KP, AC, LV, JF, and DM interpreted the data for the work. LM, DT-W, MC, OS, JD, KH, JP, KS, AC, LV, JF, and DM contributed to the manuscript revision and reviewed and approved the submitted version.

## Conflict of Interest Statement

JF and DM are among the developers of the RespirAct^TM^ for MRI studies at the University Health Network, part of the University of Toronto. Thornhill Research Inc. (TRI) is a for-profit biomedical manufacturing company that was spun off from UHN. It assembles the RespirAct^TM^ on a non-profit basis to enable MRI research at UHN and around the world. JF receives income for work done for TRI and DM holds a minor equity position in TRI. The remaining authors declare that the research was conducted in the absence of any commercial or financial relationships that could be construed as a potential conflict of interest.

## Footnotes

^1^http://www.mathworks.com/products/matlab

^2^http://www.fil.ion.ucl.ac.uk/spm/software/spm8/

^3^https://fsl.fmrib.ox.ac.uk/fsl/fslwiki/
