# Supplementary material for: Cerebrovascular Resistance in Healthy Aging and Mild Cognitive Impairment
Source: Front Aging Neurosci. 2019 Apr 12;11:79. doi: 10.3389/fnagi.2019.00079 (PMC6474328; doi:10.3389/fnagi.2019.00079)
Supplement: Supplementary file 1 [file Table_1.DOCX]

**Supplementary Material**

**Table S1. Age Matched Healthy Control and MCI Patient Demographics**

|  |  | **CON (n=11)** | **MCI (n=13)** |
| --- | --- | --- | --- |
| **Age** |  | 66.1 ± 9.1 | 75.4 ± 5.1 |
| **Gender** | **Female** | 2 | 8 |
|  | **Male** | 9 | 5 |
| **Vascular Risk Factors** | **Hypertension** | 1 | 3 |
|  | **Dyslipidemia** | 1 | 2 |
|  | **Smoking** | 0 | 1 |
| **Medications** | **Antidepressant** | 0 | 1 |
|  | **Antihypertensive** | 1 | 3 |
|  | **Statin** | 1 | 1 |
|  | **Beta-Blocker** | 0 | 1 |
| **Fazekas Score** |  | 0.73± 0.79 | 1.23 ± 0.93 |
