# Supplementary material for: Cerebrovascular Resistance in Healthy Aging and Mild Cognitive Impairment
Source: Front Aging Neurosci. 2019 Apr 12;11:79. doi: 10.3389/fnagi.2019.00079 (PMC6474328; doi:10.3389/fnagi.2019.00079)
Supplement: Supplementary file 2 [file Table_2.DOCX]

Supplementary Materials

**Table S2. Cognitive Assessment**

|  | | **CON (n=6)** | **MCI (n=13)** |
| --- | --- | --- | --- |
| **Toronto Cognitive Assessment (TorCA) total** | /330 | 294(13.70) | 257.85(20.03) |
| **TorCA subscales** |  |  |  |
| **Orientation** | /12 | 11.83(0.41) | 10.69(1.03) |
| **Immediate Memory Recall** | /30 | 21.50(2.74) | 15.54(4.06) |
| **Delayed Memory Recall** | /27 | 16.50(3.62) | 9.69(5.78) |
| **Delayed Memory Recognition** | /21 | 20.33(0.82) | 17.92(2.29) |
| **Visuospatial** | /32 | 30.50(0.55) | 29.46(1.76) |
| **Working Memory/Attention/Executive Control** | /123 | 111.83(4.67) | 99.69(10.51) |

Values are represented as mean(SD). Only 6 controls had complete cognitive assessments done in our sample. Controls were average relative to the published normative mean of the TorCA [total TorCA for ages 50-89 is 292.8(18.4) (Freedman et al., 2018)] where the MCI group fell 1.9 SD below the mean.
